# Autism Spectrum Disorders in Relation to Distribution of Hazardous Air Pollutants in the San Francisco Bay Area

**DOI:** 10.1289/ehp.9120

**Published:** 2006-06-21

**Authors:** Gayle C. Windham, Lixia Zhang, Robert Gunier, Lisa A. Croen, Judith K. Grether

**Affiliations:** 1 Division of Environmental and Occupational Disease Control, California Department of Health Services, Richmond, California, USA; 2 Impact Assessment, Inc., La Jolla, California, USA; 3 Kaiser Permanente Medical Care Program Division of Research, Oakland, California, USA

**Keywords:** air toxics, autism, autism spectrum disorders, diesel, mercury, metals, neurodevelopment, neurotoxicants, solvents, vinyl chloride

## Abstract

**Objective:**

To explore possible associations between autism spectrum disorders (ASD) and environmental exposures, we linked the California autism surveillance system to estimated hazardous air pollutant (HAP) concentrations compiled by the U.S. Environmental Protection Agency.

**Methods:**

Subjects included 284 children with ASD and 657 controls, born in 1994 in the San Francisco Bay area. We assigned exposure level by census tract of birth residence for 19 chemicals we identified as potential neurotoxicants, developmental toxicants, and/or endocrine disruptors from the 1996 HAPs database. Because concentrations of many of these were highly correlated, we combined the chemicals into mechanistic and structural groups, calculating summary index scores. We calculated ASD risk in the upper quartiles of these group scores or individual chemical concentrations compared with below the median, adjusting for demographic factors.

**Results:**

The adjusted odds ratios (AORs) were elevated by 50% in the top quartile of chlorinated solvents and heavy metals [95% confidence intervals (CIs), 1.1–2.1], but not for aromatic solvents. Adjusting for these three groups simultaneously led to decreased risks for the solvents and increased risk for metals (AORs for metals: fourth quartile = 1.7; 95% CI, 1.0–3.0; third quartile = 1.95; 95% CI, 1.2–3.1). The individual compounds that contributed most to these associations included mercury, cadmium, nickel, trichloroethylene, and vinyl chloride.

**Conclusions:**

Our results suggest a potential association between autism and estimated metal concentrations, and possibly solvents, in ambient air around the birth residence, requiring confirmation and more refined exposure assessment in future studies.

Autism is a serious neurodevelopmental disorder characterized by impairments in social interaction, verbal and nonverbal communication, and other restricted behaviors. The number of children reported with autistic spectrum disorders (ASDs) has increased dramatically during the last 10 years, but it is difficult to determine how much of this increase represents actual incidence and how much may be due to increased awareness and diagnosis; the causes remain largely unknown (Barbaresi et al. 2005; Croen et al. 2002a, 2002b; Newschaffer et al. 2005; Yeargin-Allsopp et al. 2003). Autism is believed to result from disruption of normal neurobiologic mechanisms primarily in the pre-natal period and is widely recognized to have a strong genetic component, probably involving multiple gene loci. Nongenetic factors are also likely involved and may explain some of the increased prevalence. Medications such as thalidomide and valproic acid *in utero* have been linked to cases of autism (Moore et al. 2000; Rodier and Hyman 1998; Stromland et al. 1994). Maternal smoking during pregnancy has also been associated (Hultman et al. 2002), and there are case reports of children with both fetal alcohol syndrome and autism (Aronson et al. 1997). Other exogenous exposures known or suspected to interfere with neurodevelopment may also play a role in ASD etiology. Heavy metals such as lead and mercury have been relatively well studied in relation to impaired neurodevelopment (Bellinger et al. 1984; Burbacher et al. 1990; Grandjean et al. 1997; Mendola et al. 2002), but few studies have examined associations with autism. Compounds that interfere with the endocrine system may also play a role, particularly those affecting maternal thyroid hormones, which are critical to fetal brain development (Brouwer et al. 1998; London and Etzel 2000). In addition, prenatal exposure to some solvents has recently been associated with developmental delays in offspring (Laslo-Baker et al. 2004).

Hazardous air pollutants (HAPs), as defined by the Clean Air Act Amendments of 1990, are compounds associated with adverse health outcomes such as cancer and neurologic and developmental effects [U.S. Environmental Protection Agency (EPA) 1994]. For the most part, monitoring data on these pollutants have been limited. Therefore, the U.S. EPA developed a nationwide database with modeled annual average concentrations of HAPs (Rosenbaum et al. 1999). The estimated concentrations for several compounds, including some metals, exceed the health-based benchmark concentrations for chronic toxicity in both California and the United States (Morello-Frosch et al. 2000; Woodruff et al. 1998).

To track prevalence rates of autism and to provide descriptive data on the condition, surveillance has been instituted in several states. Coordinated by the Centers for Disease Control and Prevention (CDC), these programs have been organized into Centers for Autism and Developmental Disabilities Research and Epidemiology (CADDRE) and Autism and Developmental Disorders Monitoring (Rice et al. 2004; Yeargin-Allsopp et al. 2003). In six counties in the San Francisco Bay area, we are conducting multi-source surveillance to ascertain ASD cases identified from clinical sources as well as from the Department of Developmental Services (DDS), which provides services for California residents with a variety of eligible developmental disabilities.

We conducted an exploratory case–control analysis linking our autism surveillance data to HAPs data for the San Francisco Bay area to examine the potential role of ambient chemical exposures during pregnancy or early life in ASD etiology.

## Materials and Methods

### Subjects

This study was approved by the California Committee for the Protection of Human Subjects. The population of interest included children born in 1994 to mothers resident at delivery in one of six San Francisco Bay area counties (Alameda, Contra Costa, Marin, San Francisco, San Mateo, and Santa Clara), representing approximately 80,000 births. Children with ASD were identified through the active surveillance conducted by California CADDRE, representing an approximate population-based series of affected children identifiable from existing records. At the time this study was conducted, the sources for case ascertainment were the DDS and the Kaiser Permanente Medical Care Program. Previous work has shown that DDS probably serves 75–80% of children with autistic disorder, or those on the more severe end of the autism spectrum (Croen et al. 2002a). From the DDS electronic database, California CAD-DRE staff originally identified clients with a diagnosis of autism or with mental retardation, epilepsy, or other developmental disorder with no known cause, before the child’s ninth birthday. DDS records statewide were linked to birth certificate records to identify any cases born to mothers living in the six-county study area. Approximately 25% of births in these counties occur among Kaiser members, who are generally representative of the population except for the extreme ends of socioeconomic status (SES) (Krieger 1992). From Kaiser electronic files, children with an ASD diagnosis [Diagnostic and Statistical Manual of Mental Disorders, 4th ed. (DSM-IV; 2000) code 299.00 or 299.80] before their ninth birthday were selected and then linked to birth certificates to identify resident births and duplicates with DDS. Trained CADDRE staff reviewed the DDS and Kaiser medical records of all identified children and abstracted standardized data for 341 with evidence of autism behaviors. About 21% were identified only in Kaiser, not DDS, and 25% were found in both systems. Final case status was determined by computer algorithm and several levels of expert review by a CADDRE principal investigator (J.G.) and/or a child psychiatrist with expertise in ASDs. This review process yielded 284 cases (83.3%) who met the stricter surveillance definition of ASD, having at least one of the following: *a*) a diagnosis of ASD from a qualified medical professional, *b*) qualification for special education under an autism exceptionality, or *c*) autistic behaviors that appear to meet DSM-IV criteria for a diagnosis of autistic disorder, Asperger’s, or Pervasive Developmental Disorder not otherwise specified, per expert review.

We randomly selected control children for this study from the California 1994 linked birth–infant death certificate file with maternal residence at delivery in one of the six counties, matched to the original cases in a ratio of two to one by sex and month of birth (*n* = 682 for original cases). We subsequently excluded known deaths as well as controls served by DDS under other diagnoses (*n* = 8). We abstracted birth addresses from hard copies of the birth certificates, but several records were sealed because of adoption (*n* = 7). Demographic data and infant characteristics were obtained from the birth certificate.

We geocoded the birth addresses to obtain census tract for linkage to HAPs data. Using ArcGIS (version 9.0; ESRI Inc., Redlans, WA) and GDT version 11.1 street data for 2001 (Geographic Data Technology, Inc., Lebanon, NH), 95% were successfully geocoded via batch processing. The remaining 5% were manually geocoded. We then assigned a 1990 census tract based on the street segment where the geocoded addresses were located and the census tract boundaries (using Dynamap 2000 version 11.1 from GDT). Ten control addresses were not successfully assigned a tract, leaving 284 cases and 657 controls for our analysis.

### Exposure assessment: hazardous air pollutant concentrations

The U.S. EPA estimates HAPs concentrations using a Gaussian air dispersion model that combines emissions inventories from mobile, point, and area sources with data on local meteorology, chemical decay rates, secondary formation, and deposition (Rosenbaum et al. 1999; Woodruff et al. 1998). Mobile sources include motor vehicles, airplanes, trains, and ships, whereas area sources include emissions from smaller stationary sources such as dry cleaners, gas stations, and residential use of products, and point sources are large industrial manufacturing facilities. Estimated concentrations are summed across these sources and background levels from “clean air locations” are added. Annual average HAPs concentration estimates are available at the U.S. census tract level for 1990 and 1996. We used the 1996 data because they were closer to the birth year of the subjects, and improvements had been made since 1990 in the emissions inventory data and the assumptions used in the dispersion model (U.S. EPA 2002a).

Because little prior information indicated which of the 33 compounds in the 1996 database might be related to autism, we took a broad approach, examining compounds that are recognized developmental toxicants or suspected neurologic toxicants and endocrine disruptors [California Environmental Protection Agency (CalEPA) 2003, 2005; Colborn et al.1993; Illinois Environmental Protection Agency (ILEPA) 1997; Keith 1997; National Institute for Occupational Safety and Health (NIOSH) 2001; U.S. EPA 2003]. We also considered chemicals that had been identified as contaminants of concern for an autism cluster investigation [Agency for Toxic Substances and Disease Registry (ATSDR) 2000], which resulted in adding only one chemical (chromium). We examined diesel particulate matter, although it does not specifically meet the above criteria, because diesel exhaust contains several compounds with relevant toxicity for autism, including arsenic, benzene, nickel, and polyaromatic hydrocarbons (PAHs). Thus, we examined 25 compounds with some toxicity potentially relevant to autism (Table 1).

We found that six compounds (Table 1) had a poor distribution and very little variability across the 1,228 census tracts in the study area, so we excluded them from further analyses. The concentrations of many of the remaining 19 compounds were highly correlated: 11 had Spearman correlation coefficients of ≥ 0.85 with more than one other compound. Because of the difficulty inherent in evaluating separate effects of these correlated compounds, we examined them in groups. Given toxicologic evidence, we grouped the compounds by mechanistic properties into developmental toxicants (*n* = 7) and endocrine disruptors (*n* = 10), which include some compounds in common (Table 1). We also grouped the compounds by structural properties into metals (*n* = 7), aromatic solvents (*n* = 5), and chlorinated solvents (*n* = 4), which are mutually exclusive (Table 1).

The mean concentrations of the compounds within a group varied by orders of magnitude (Table 1), so summing them to obtain an overall concentration for the group would underrepresent exposure to the chemicals with lower means. Therefore, we calculated an index score for each group. First, we categorized into quartiles the distributions of each individual compound across the census tracts in which controls were born. Then we assigned a level of one to four based on the quartile (low–high) and summed across the compounds included in each group to obtain an overall score for that chemical group, for each census tract. For example, with seven metals in the heavy metal group, the range of possible scores for a census tract was (7 × 1, if all low levels) to (7 × 4, if all high), or 7–28. The census tract group score was assigned to all cases and controls born in that tract. The mid-point of the score (e.g., 18 in the example above) generally corresponded well with the median of the score distribution, but because the distributions were non-normal, we categorized the scores into quartiles. We examined individual chemicals categorically as well, using the quartile cut points determined from the control distribution.

### Statistical analyses

To maintain as large a sample size as possible, we did not exclude controls (*n* = 114) whose matched cases did not meet the surveillance criteria. Univariate analyses included examining quartile levels of chemicals and chemical groups described above by case–control status. For descriptive purposes, we also compared means of individual compounds by case–control status. We examined the potential covariates (maternal age, race, education, and parity; paternal race and age; low birth weight, preterm delivery, and child race) as categorical variables by the quartiles of the chemical group scores, as well as by case–control status. We included those associated with chemical exposure as well as case status and those not highly redundant, such as parental and child race, in the final logistic regression models; these were child race, maternal age, and maternal education. The original matching variables did not meet these criteria, but we checked the effect of adding them to the models for the chemical groups; because it made little difference in the results, we did not maintain them in the final models. For the models, we calculated dummy variables for the third and fourth quartile exposure levels and combined the lower two quartiles as the referent group (below the median) to increase power and because there were generally not effects at the second quartile level. In some regression models we also included more than one chemical, or chemical group if they were mutually exclusive, to adjust for each other. Because a strict case–control match was not maintained, we did not use conditional logistic regression modeling except as a check on the findings from logistic regression models.

## Results

Compared with controls, cases were somewhat more likely to be white and less likely to be Hispanic, and to be born to mothers who were somewhat older and better educated (Table 2). This pattern also held for paternal age and education. The male:female ratio was 4:1, as expected from previous work (Croen et al. 2002b). Some of the demographic variables varied by exposure level, with nonwhites and younger and less-educated parents generally more likely to live in areas with higher exposure concentrations of both metals and solvents (data not shown).

The aromatic solvents and diesel particulate matter had the highest concentrations among the HAPs we examined (Table 1). The compounds with the widest range of concentrations among controls (e.g., standard deviation equal to or greater than the mean) tended to be the metals, as well as vinyl chloride and hydrazine (Table 1). The crude mean levels of the individual compounds were generally similar or slightly higher in cases compared with controls, particularly for diesel particulate matter and toluene (Table 1).

In logistic regression models that included a single chemical group, the adjusted odds ratios (AORs) for the mechanistic groups were slightly elevated for the fourth quartile levels (1.3 for endocrine disruptors and 1.4 for developmental toxicants (Table 3). By structural groups, AORs were elevated about 50% for fourth quartile levels of metals and chlorinated solvents (Table 3), and the AOR was also elevated for the third quartile level of metals. In models that adjusted for these groups together, the AORs were reduced for the solvents but were slightly higher for the metal group [metal AOR = 1.95; 95% confidence interval (CI), 1.23–3.09, and AOR = 1.74; 95% CI, 1.01–3.01 for the third and fourth quartile levels, respectively].

We looked further at the metal and chlorinated solvent groups to identify whether the observed associations were for the group in general or linked to specific compounds (Table 4). Among the chlorinated solvents, AORs for several compounds were slightly elevated at the third quartile, and AORs for trichloroethylene and vinyl chloride were significantly elevated at the fourth quartile (AORs = 1.47 and 1.75, respectively). Among the metals, cadmium, mercury, and nickel had elevated AORs for the fourth quartiles (Table 4). Diesel particulate matter was examined separately and showed a similar magnitude of association (AOR = 1.44; 95% CI, 1.03–2.02). Diesel particulate matter, mercury, trichloroethylene and vinyl chloride showed elevated odds ratios (ORs) at the 90th percentile category as well (AORs = 1.6–1.9, data not shown).

As noted earlier, some of these compounds were strongly correlated to each other. Vinyl chloride was the least correlated with other compounds but showed some correlation with mercury (*r* = 0.70) and cadmium (*r* = 0.58), which were themselves correlated (*r* = 0.76). Nickel was most correlated with arsenic (*r* = 0.86) and cadmium (*r* = 0.77), and trichloroethylene was correlated with all three of these metals (*r* ≥ 0.77). Concentrations of diesel particulate matter were also somewhat correlated with a few metals (*r* = 0.77–0.79, namely, arsenic, cadmium, and mercury). The aromatic solvents were all highly correlated with one another (*r* = 0.89–0.99) as well as to PAHs and manganese. We attempted to separate the mercury/cadmium relationship further by including both of these in one model; the AOR for the fourth quartile of mercury remained elevated (2.1; 95% CI, 1.25–3.50), but that for cadmium was reduced to below one. We examined their joint distribution by comparing subjects that had concentrations above the median for both compounds, or above the median for just one, with those with concentrations of both that were at or below the median. After adjustment, the AOR for the category of higher levels of both remained elevated at 1.75 (95% CI, 1.25–2.45), and the AORs for higher levels of either cadmium or mercury alone were in a similar range (AOR = 1.31; 95% CI, 0.77–2.25 and AOR = 1.55; 95% CI, 0.96–2.52, respectively). A similar analysis of mercury and vinyl chloride yielded AORs that were greatest for the higher mercury-only category (AOR = 2.04; 95% CI, 1.27–3.28), but in a similar range as higher vinyl chloride only (1.56, 95% CI, 0.95–2.56), or higher for both (AOR = 1.74; 95% CI, 1.24–2.45).

## Discussion

These data suggest a potential association of autism with higher ambient air concentrations of metals and possibly chlorinated solvents in the geographic area of birth residence. There are several limitations to the exposure data to consider. Concentrations of many chemicals were correlated, so it was difficult to untangle specific chemicals of interest. Therefore, we combined levels of structurally similar chemicals using an index score similar to one used by others to examine mixtures (Swan et al. 2005). The concentrations represent modeled estimates of outdoor air levels based on chemical emissions in a geographic area, not actual personal measurements. The estimates used do not take into account mobility or specific maternal activities during pregnancy or child activities post-natally. Measurement studies have shown that personal exposures to volatile organic compounds (including the solvents) typically exceed measured outdoor air concentrations (Adgate et al. 2004; Sax et al. 2004), but that the U.S. EPA 1990 modeled HAPs concentrations were reasonable surrogates for personal exposure (Payne-Sturges et al. 2004). In general, the 1996 modeled estimates for most of the pollutants underestimate the measured ambient concentrations available from limited monitoring stations, particularly for the metals, although mercury was not examined (U.S. EPA 2002a). Our subjects were actually born in 1994, not in 1996 when the estimates were made, but based on available air monitoring data (California Air Resources Board 2005), it is unlikely that the relative rank of concentrations varied greatly in such a short time. Furthermore, we do not have addresses for the first trimester of pregnancy, which may be of most concern etiologically. Finally, the exposure estimates do not include other sources of chemical exposure such as occupational, active or passive smoking, or (particularly for metals) diet. These limitations lead to misclassification of exposure, but as this is unlikely to vary by case status, the effect estimates are probably shifted toward the null. Despite these limitations of the exposure data, the HAPs database has been used to investigate associations with other health outcomes, including childhood cancer (Reynolds et al. 2003) and reproductive outcomes (Vassilev et al. 2001).

This study had other minor limitations, including information on potential covariates available only from the birth certificate. However, several do reflect SES (e.g., education and race). These were considered likely *a priori* confounders because HAPs concentrations tend to be higher in lower SES census tracts (Morello-Frosch et al. 2002), whereas autism may be more likely to be detected among higher SES groups. Because so little is known about risks for autism, it is possible that uncontrolled confounding may partly explain the observed associations; for example, we had no data on maternal conditions or habits. The cases included in this study likely represent more severely affected children because of the nature of our case ascertainment sources. These children would be less likely to have diagnosis dependent on access and parental means. However, if children of lower SES who are more likely to be exposed were underrepresented in the case group, this could decrease the magnitude of effects observed.

Strengths of the study include availability of valid sources for identifying a population-based sample of cases and confirmation of diagnosis by review of records. Linkage to existing environmental exposure databases fulfills the mission of environmental health tracking programs, allowing relatively inexpensive study of retrospective exposure, which is not affected by recall bias. Examining 1990 HAPs levels in California, one study indicated that the urban areas, including the San Francisco Bay area, had the highest levels compared with other counties (Morello-Frosch et al. 2000), perhaps improving likelihood of detecting an association in this study. Although we examined many compounds, they were selected *a priori*, and the number with statistically significant associations was far greater than would be expected by chance. Our results were robust across various reanalyses of the data that included a less restrictive case definition or reassignment of census tract and exposure level, as well as when analyzed by conditional logistic regression using only individually matched controls.

There is limited prior work on environmental exposures that may be associated with autism in humans, but some plausibility for effects (reviewed by Allred and Wilbur 2002; Lawler et al. 2004; London and Etzel 2000). Prior studies have reported associations of autism with maternal smoking (Hultman et al. 2002), heavy alcohol consumption (Aronson et al. 1997), some prescription medications (Moore et al. 2000; Stromland et al. 1994), and parental occupations involving chemical exposures (reviewed by Allred and Wilbur 2002). These observations, combined with those from animal and neuroimaging studies, suggest that exposures early in gestation, around the time of neural tube closure, may be most critical (Rodier and Hyman 1998). A strong genetic component is indicated in the etiology of autism; it has been hypothesized this could involve susceptibility genes that, when combined with exposure, lead to this condition (London and Etzel 2000).

Of the postulated chemicals of interest in relation to autism, metals, particularly mercury, have generated the most attention. Several metals have been implicated in adverse neurodevelopmental outcomes in children, notably lead and mercury (ATSDR 1999a; Bellinger et al. 1984; Counter and Buchanan 2004; Mendola et al. 2002), with exposure to cadmium, arsenic, and chromium also of concern. Studies have found adverse effects of prenatal lead exposure on growth and development (Dietrich 1991), but little research has examined an association with autism (Eppright et al. 1996). Mercury is of concern because of evidence for neurotoxic effects and the fact that it has become ubiquitous in the global environment (Counter and Buchanan 2004; National Research Council 2000). Elemental mercury, released into the environment from the erosion of ores, industrial fossil fuel emissions (e.g., coal burning for power), and industrial waste, is the form of mercury represented in the HAPs database. The highest environmental exposure to mercury in humans currently is from methylmercury in the diet, but there is little study related to autism. Several incidents of widespread methyl mercury poisoning decades ago resulted in serious neurodevelopmental impairments in prenatally exposed children (Bakir et al. 1973; Tsubaki and Irukayama 1977). Ethylmercury, used in medical products and as a preservative (thimerosal) in common vaccines, contributes to total mercury levels in the blood, but there is little direct evidence of health effects, and expert reviews have concluded that vaccines are not associated with autism (Heron et al. 2004; Institute of Medicine 2004; Parker et al. 2004). Thimerosal has been removed from routine pediatric vaccines, but public debate and animal research continue (Burbacher et al. 2005; Geier and Geier 2003). Studies in animals have shown effects of elemental mercury that appear comparable to methylmercury or that are potentiated by joint exposure (ATSDR 1999a; Warfvinge 2000). Prenatal or early postnatal exposure to elemental mercury resulted in subtle behavioral changes in offspring in some studies and hyperactivity and alterations in spontaneous and learned behaviors that suggested deficits in adaptive functions (ATSDR 1999a). Although these data support our findings with elemental mercury, it would be most useful to have data on personal exposure to all forms of mercury from early pregnancy into childhood, which is logistically difficult. In addition to neurotoxic effects, some of the metals, including mercury, are suspected endocrine disruptors (Table 1), with effects on thyroid function also noted (ATSDR 1999a; Ellingsen et al. 2000; Takser et al. 2005).

A recent epidemiologic study (Palmer et al. 2006) linking Toxic Release Inventory (TRI) data on mercury to special education data in Texas reported a 61% increase in autism prevalence rates (or 17% adjusted) per 1,000 pounds of mercury released. The TRI industrial mercury emissions data are included as input data (from point sources) in the more complex model calculating HAPs concentrations that we used. Further interpretation and comparison of findings between our study and the Texas study are hampered by differences in the exposure measure (point source emissions vs. total concentrations used in this study), geographic scale (large counties vs. census tracts ), and time period (year of school enrollment vs. year of birth).

Like mercury, cadmium is a recognized developmental toxicant with adverse effects on fetal growth and perhaps fetal viability at high doses (CalEPA 2005). There are few human data on neurodevelopmental effects, but in animals high prenatal levels were associated with changes in behavior and learning ability in offspring (ATSDR 1999b). Cadmium is also a suspected endocrine disruptor, with effects on steroidogenesis observed (Henson and Chedrese 2004).

Our results for aromatic solvents are difficult to interpret because the concentrations of these solvents were highly intercorrelated and tended to show less variation across the geographic area. We found moderate associations of autism with higher chlorinated solvent concentrations. These were lessened in models that adjusted for metals as well, but this could reflect some overadjustment. Vinyl chloride had the largest ORs of the chlorinated solvents and was not highly correlated to the others. Maternal solvent exposure has been associated with various adverse pregnancy outcomes, including spontaneous abortion, intrauterine growth retardation, and congenital malformations such as neural tube defects (ATSDR 1998; Bove et al. 1995; Cordier et al. 1997; McMartin et al. 1998; Windham et al. 1991; reviewed by Windham and Osorio 2004). A recent study followed offspring of women occupationally exposed to organic solvents and found that compared with unexposed children, these children obtained lower scores on subtests of intellectual, language, motor, and neurobehavioral functioning (Laslo-Baker et al. 2004). Together with our results, these suggest solvents should be examined further in relation to autism.

The moderate association we found with higher diesel particulate matter levels may in part be due to some correlation with metals. Nevertheless, studies of reproductive outcomes in New Jersey found the highest tertile level of airborne polycyclic organic matter, a related class of particulate matter, was associated with risks increased 20–30% for preterm birth, low birth weight, and fetal death (Vassilev et al. 2001). Results of a study of diesel exhaust exposure in neonatal rats suggested permanent alterations in both learning ability and activity, indicating that the significance to humans should be pursued further (U.S. EPA 2002b). Other animal studies have indicated potential endocrine-disrupting effects of prenatal exposure to diesel exhaust (Watanabe and Kurita 2001) and increased indices of inflammation in brains of mice exposed to airborne particulate matter (Campbell et al. 2005).

Environmental exposures occur in mixtures determined by emissions sources, so it is difficult to disentangle effects of specific compounds or groups of compounds, and adverse health effects may be potentiated by joint exposures. However, when we examined joint exposure of mercury with cadmium or vinyl chloride, clear interaction was not noted. Within the six counties we studied, San Francisco County had by far the highest mean levels for six representative compounds we compared (mercury, cadmium, diesel particulate matter, methylene chloride, toluene, and vinyl chloride) and also had a higher ratio of cases to controls than overall (0.71 vs. 0.43). In contrast, Marin County, with the lowest levels of these chemicals, had a much lower ratio (0.14). However, these patterns may reflect other factors, including diagnostic differences or care-seeking behavior.

## Conclusions

Results of this semiecologic study suggest that living in areas with higher ambient levels of HAPs, particularly metals and chlorinated solvents, during pregnancy or early childhood, may be associated with a moderately increased risk of autism. These findings illuminate the need for further scientific investigation, because although potentially biologically plausible they are preliminary and require confirmation. The autism surveillance network funded by the CDC and the availability of HAPs data nationwide provide the opportunity for similar linkage studies to be conducted in other locations, and we plan to look at 1996 autism surveillance data when available. Additional sources or refinement of such data may be available in different states or regions and could also be examined. More complex etiologic studies with measurements of individual level exposures to multiple compounds by various pathways (air, water, diet), combined with genetic information, will be important to further our understanding of the potential contribution of environmental exposures to the development of autism.

## Figures and Tables

**Table 1:** Classification and distribution of concentrations of HAPS potentially relevant to autism.

				Mean ± SD (μg/m^3^)	
Chemical groups	Suspected neurologic toxicant^a^	Recognized developmental toxicant^b^	Suspected endocrine toxicant^c^	Cases	Controls
Metals					
Arsenic^d^	X	X	X	0.0001 ± 0.00006	0.0001 ± 0.00005
Cadmium^d^	X	X	X	0.0001 ± 0.0002	0.0001 ± 0.0001
Chromium^d^				0.0044 ± 0.0057	0.0039 ± 0.0049
Lead^d^	X	X	X	0.0093 ± 0.0118	0.0082 ± 0.0092
Manganese	X			0.0032 ± 0.0017	0.0032 ± 0.0016
Mercury^d^	X	X	X	0.0008 ± 0.0019	0.0006 ± 0.001
Nickel	X			0.0043 ± 0.0059	0.0037 ± 0.0038
Aromatic solvents					
Benzene^d^	X	X	X	1.71 ± 0.62	1.66 ± 0.50
Ethyl benzene^d^	X		X	0.94 ± 0.44	0.91 ± 0.38
Styrene	X		X	0.10 ± 0.06	0.09 ± 0.05
Toluene^d^	X	X		6.98 ± 4.08	6.44 ± 3.00
Xylene^d^	X	X		3.77 ± 1.68	3.63 ± 1.46
Chlorinated solvents					
Methylene chloride^d^	X		X	0.68 ± 0.48	0.64 ± 0.35
Perchloroethylene^d^	X			0.61 ± 0.33	0.60 ± 0.34
Trichloroethylene^d^	X			0.19 ± 0.11	0.17 ± 0.08
Vinyl chloride^d^	X			0.02 ± 0.06	0.01 ± 0.02
Other HAPs					
Hydrazine	X		X	1.29 ×10^−7^ ± 2.96 ×10^−7^	1.16 ×10^−7^ ± 2.39 ×10^−7^
PAHs (7)^d^			X	0.0085 ± 0.0042	0.0086 ± 0.0041
Diesel PM^e^				3.37 ± 3.48	2.89 ± 2.35
Poor distributions^f^					
Carbon tetrachloride^d^	X		X	—	—
Chloroform^d^	X		X	—	—
Ethylene dibromide	X	X	X	—	—
Ethylene dichloride	X		X	—	—
Hexachlorobenzene	X	X	X	—	—
PCBs^d^	X	X	X	—	—

Abbreviations: PCBs, polychlorinated biphenyls; PM, particulate matter.

a Suspected neurologic toxicants (ATSDR 2000; CalEPA 2003; NIOSH 2001; U.S. EPA 2003).

b Recognized developmental toxicants (CalEPA 2005).

c Suspected endocrine disruptors (Colburn et al. 1993; ILEPA 1997; Keith 1997; NIOSH 2001).

d Also on list of contaminants of concern for autism from ATDSR Brick Township Investigations (ATSDR 2000).

e Diesel PM included because it contains compounds on the list including arsenic, benzene, nickel, and PAHs.

f There was very little variability in estimated concentrations across most census tracts in study area, so these were excluded.

**Table 2:** Demographic characteristics of autism cases and live born–controls born in San Francisco Bay area, 1994.

Variable	Percent of cases (*n* = 284)	Percent of controls (*n* = 657)	Chi-square *p*-value
Male sex	84.9	81.0	0.15
Child’s race			0.09
White	46.1	39.6	
Hispanic	18.1	26.3	
Other	35.8	34.1	
Maternal age (years)			0.09
< 25	19.0	25.6	
25–35	63.7	59.5	
≥ 35	17.3	14.9	
Maternal education			0.0001
< High school	9.9	17.7	
High school graduate	24.0	26.2	
Some college	33.9	21.5	
College graduate	32.2	34.6	
Parity			0.33
1	43.0	45.4	
2–3	51.1	46.6	
≥ 4	6.0	8.1	

**Table 3:** Distribution and AOR^a^ (95% CI) for autism risk by quartile^b^ of hazardous air pollutant groups.

	HAP group level		
Group^c^	First and second quartiles no. of cases/controls Referent group	Third quartile no. of cases/controls AOR (95% CI)	Fourth quartile no. of cases/controls AOR (95% CI)
Mechanistic			
Endocrine disruptors	128/328	86/173 1.33 (0.94–1.88)	70/156 1.28 (0.88–1.85)
Developmental toxicants	139/319	68/156 1.13 (0.79–1.63)	77/152 1.40 (0.98–2.00)
Structural			
Aromatic solvents	148/328	64/173 0.84 (0.59–1.20)	72/156 1.15 (0.80–1.65)
Chlorinated solvents	136/368	74/157 1.33 (0.93–1.88)	74/132 1.55 (1.08–2.23)
Metals	123/348	79/141 1.68 (1.17–2.41)	82/168 1.50 (1.05–2.12)

a Adjusted by logistic regression for maternal age, education and child race in separate models for each chemical.

b Quartile cut points determined from distribution of index score among controls.

c See text or Table 1 for definition of groups. Mechanistic groups overlap, e.g., some compounds are classified in both. Structural groups are mutually exclusive.

**Table 4:** AORs^a^ (95% CIs) for upper quartiles of metals and chlorinated solvents by autism case–control status.

	Third quartile^b^	Fourth quartile^b^
	AOR (95% CI)	AOR (95% CI)
Chemical chlorinated solvents		
Methylene chloride	1.50 (1.06–2.13)	1.37 (0.96–1.96)
Perchloroethylene	1.31 (0.93–1.84)	1.11 (0.78–1.59)
Trichloroethylene	1.37 (0.96–1.95)	1.47 (1.03–2.08)
Vinyl chloride	1.01 (0.69–1.47)	1.75 (1.25–2.43)
Metals		
Arsenic	1.07 (0.75–1.53)	1.28 (0.90–1.81)
Cadmium	1.43 (1.01–2.04)	1.54 (1.08–2.20)
Chromium	0.83 (0.58–1.20)	1.12 (0.79–1.58)
Lead	0.75 (0.52–1.09)	1.07 (0.76–1.51)
Manganese	1.12 (0.79–1.58)	1.09 (0.75–1.59)
Mercury	1.31 (0.91–1.88)	1.92 (1.36–2.71)
Nickel	1.11 (0.77–1.59)	1.46 (1.04–2.06)

a Adjusted by logistic regression for maternal age, education, and child race in separate models for each chemical. Reference is median or less.

b Quartile cut points determined from distribution among controls.
